# Comparability and Validity of the Online and In-Person Administrations of the Inventory of Problems-29

**DOI:** 10.1007/s12207-021-09406-0

**Published:** 2021-04-05

**Authors:** Luciano Giromini, Claudia Pignolo, Gerald Young, Eric Y. Drogin, Alessandro Zennaro, Donald J. Viglione

**Affiliations:** 1 Department of Psychology, University of Turin, Via Verdi 10, 10123 Torino, TO Italy; 2 Glendon College, York University, Toronto, Canada; 3 Department of Psychiatry, Harvard Medical School, Boston, MA USA; 4 Alliant International University – San Diego, San Diego, CA USA

**Keywords:** Teleassessment, Malingering, Symptom validity assessment, IOP-29, Online

## Abstract

While the psychometric equivalence of computerized versus paper-and-pencil administration formats has been documented for some tests, so far very few studies have focused on the comparability and validity of test scores obtained via in-person versus remote administrations, and none of them have researched a symptom validity test (SVT). To contribute to fill this gap in the literature, we investigated the scores of the Inventory of Problems-29 (IOP-29) generated by various administration formats. More specifically, Study 1 evaluated the equivalence of scores from nonclinical individuals administered the IOP-29 remotely (*n* = 146) versus in-person via computer (*n* = 140) versus in-person via paper-and-pencil format (*n* = 140). Study 2 reviewed published IOP-29 studies conducted using remote/online versus in-person, paper-and-pencil test administrations to determine if remote testing could adversely influence the validity of IOP-29 test results. Taken together, our findings suggest that the effectiveness of the IOP-29 is preserved when alternating between face-to-face and online/remote formats.

The recent COVID-19 pandemic has greatly disrupted the professional routine of clinical psychologists and neuropsychologists who provide assessment services and mental health evaluations. Given the necessity of physical distancing, several major assessment instruments developed guidelines to help in the switch from direct face-to-face interaction to remote assessment (American Psychological Association, 2020; Chenneville & Schwartz-Mette, 2020; Farmer et al., 2020; Pliskin et al., 2020; Wright et al., 2020). However, while the psychometric equivalence of computerized versus paper-and-pencil administration formats has been documented for some of these measures (e.g., MMPI-2/MMPI-2-RF: Finger & Ones, 1999; Forbey & Ben-Porath, 2007; Menton et al., 2019; Pinsoneault, 1996; Roper et al., 1995; WISC-V: Daniel et al., 2014, Daniel & Wahlstrom, 2019), to date, very few studies have focused on the possible differences between remote and face-to-face administration methods (e.g., personality inventories: Chuah, 2006; intelligence and achievement tests: Wright, 2018; neuropsychological tests: Brearly et al., 2017; Marra et al., 2020). This gap in the literature is even more evident when one considers the validity of computerized and/or remote administration of tests that evaluate the credibility of reported symptoms and response styles (Kois et al., 2020).

Within the field of forensic mental health evaluations, some authors (Carroll, 2020; Drogin, 2020; Kois et al., 2020; Levy, 2020) have argued that it is only a matter of time before the courts will engage in a legal debate on whether teleassessment represents a significant and problematic departure from standard testing protocols. Indeed, as stated by the Joint Task Force for the Development of Telepsychology Guidelines for Psychologists (2013) “when a psychological test or other assessment procedure is conducted via telepsychology, psychologists are encouraged to ensure that the integrity of the psychometric properties of the test or assessment procedure (e.g., reliability and validity) and the conditions of administration indicated in the test manual are preserved when adapted for use with such technologies” (p. 798). Put differently, psychological tests that have yet to be ascertained as reliable and valid in the remote testing context should be established as such before their administration in remote forensic testing. The need for research on these questions should therefore rank high on the research agenda of academics and practitioners alike.

Our article responds to this call for research by focusing on the comparability and validity of the remote and in-person administrations of a brief, self-administered symptom validity test (SVT): the Inventory of Problems-29 (IOP-29; Viglione & Giromini, 2020). Study 1 empirically investigated the extent to which nonclinical individuals administered the IOP-29 remotely (online) versus in-person via computer versus in-person via paper-and-pencil format produced similar test results. Study 2 reviewed published IOP-29 studies conducted using remote/online versus in-person, paper-and-pencil test administrations to compare the data on different administration methods in order to determine if remote testing could adversely influence the validity of IOP-29 test results.

## The Inventory of Problems-29 (Viglione & Giromini, 2020)

The IOP-29 is a brief SVT designed to assist practitioners making determinations on whether a given clinical presentation is more likely to be either bona fide or feigned. It is applicable to a wide range of conditions involving depressive, psychotic, neurocognitive, and/or PTSD-related psychological problems. Comprised of 29 items, it is typically completed within < 10 min. Twenty seven of the IOP-29 items are self-report, SVT-like statements or questions that offer three response options: “True,” “False,” and “Doesn’t make sense.” The remaining two items are cognitive (e.g., calculation, logic) problems that call for open-ended responses. According to Viglione et al. (2017), this set of 29 items survived multiple cross-validations throughout a lengthy period of test development in which an initial pool of 245 items addressing 27 feigning strategies were evaluated. This procedure is probably one of the reasons why, despite its brevity, the IOP-29 seems to be a highly effective SVT (Gegner et al., 2021; Giromini et al., 2018; Ilgunaite et al., 2020; Roma et al., 2020; Viglione et al., 2017; Winters et al., 2020).

Its feigning score, named False Disorder Score (FDS), is a probability score that ranges from 0 to 1: the higher the FDS, the lower the credibility of the presented complaint(s). According to the test manual (Viglione & Giromini, 2020), a cutoff score of FDS ≥ 0.50 likely offers the best balance between sensitivity and specificity, yielding an average classification accuracy of about 80% (see also Giromini et al., 2018). To reach a specificity level of about 90%, which is typically suggested in high-stake assessments (Larrabee, 2003), a more suitable cutoff would be FDS ≥ 0.65. In contrast, in a screening context, in which sensitivity should be favored over specificity, an optimal cutoff score would be FDS ≥ 0.30 so to reach a sensitivity level of about 90% (Viglione & Giromini, 2020). Below we summarize some of the distinctive features of the IOP-29 and the research foundation for using it in applied settings.

### Distinctive Features of the IOP-29

Several distinctive features characterize the IOP-29, making it different from all other extant SVTs. Three of the most relevant ones are as follows. First, while the typical SVT mainly (if not only) relies on the rare-symptoms endorsement detection strategy (Rogers & Bender, 2018), the IOP-29 uses multiple detection strategies, including some derived from interviewing techniques (Viglione et al., 2017). Thus, rather than exclusively focusing on whether or not a test-taker suffers from a series of uncommon or non-existent symptoms, the IOP-29 also investigates how examinees cope with their problems—if they believe there is anything they can do to lessen the burden their problems generate, if they take any responsibility for their difficulties, and so forth. Second, as noted above, in addition to the classic “True” and “False” response options, the SVT-like items of the IOP-29 also offer a third option, “Doesn’t make sense.” In the studies leading up to the final refinements and release of the test, Viglione et al. (2017) found that this third response option psychometrically improved the overall signal detection of almost all IOP-29 items, compared to using the standard True/False dichotomy. Additionally, it also contributed to the assessment of possible resistance to the evaluation and/or feigned cognitive deficiency. Third, the feigning score of the IOP-29 is not based on a single set of normative reference data obtained from healthy volunteers, as is the case for many other SVTs. To allow a more precise determination of the likelihood of a given presentation to be “valid” versus “invalid,” the IOP-29 FDS indeed compares the test-taker’s responses against two different sets of reference values, one coming from bona fide patients, and the other one coming from experimental simulators. This is done by implementing a logistic regression derived, exponential function that generates a probabilistic score reflecting the likelihood of obtaining a given IOP-29 from either one of the two aforementioned sets of reference values. From a practical standpoint, this technical innovation is deemed “to assist and simplify the decision-making process of mental health professionals performing symptom and/or performance validity assessment” (Viglione & Giromini, 2020, p. 43).

The fact that the IOP-29 is so different from typical SVTs makes it particularly suitable to be included in a multi-method symptom validity assessment. Indeed, as it does not primarily rely on the rare-symptoms endorsement detection strategy, the IOP-29 likely offers valuable, complementary information (and thereby incremental validity) when used in combination with other useful SVTs such as the F scales of the Minnesota Multiphasic Personality Inventory (MMPI-3; Ben-Porath & Tellegen, 2020a, b), the Structured Inventory of Malingered Symptomatology (SIMS; Smith & Burger, 1997), or the Self-Reported Symptom Inventory (SRSI; Merten et al., 2016). In support of this hypothesis, a recent clinical comparison simulation study focused on depression-related complaints found that using the IOP-29 together with the *F* scales of the MMPI-2 (Butcher et al., 2001), in fact, provided statistically significant incremental validity over using either instrument alone (Giromini et al., 2019).

### Research Foundation

As noted by the Editor-in-Chief of Psychological Injury and Law and his colleagues in a recent article aimed at introducing the field of psychological injury and law, the IOP-29 is “a newer stand-alone SVT that has the required psychometric properties for use in forensic disability and related assessments. Its research profile is accumulating, a hallmark for use in legal settings” (Young et al., 2020, p. 9). Although the IOP-29 was published only relatively recently in 2017 (Viglione et al., 2017), all 12 published studies since then support its validity and effectiveness (Gegner et al., 2021; Giromini et al., 2018, 2020a, b, c, d; Ilgunaite et al., 2020; Roma et al., 2020; Viglione et al., 2017, 2019; Winters et al., 2020). Specifically, the results of these studies suggest that (a) the validity and classification accuracy of the IOP-29 compares favorably to that of popular measures like the Structured Inventory of Malingered Symptomatology (SIMS; Smith & Burger, 1997) (Giromini et al., 2018) or Rey Fifteen-Item Test (FIT; Lezak, 1995; Rey, 1941) (Gegner et al., 2021); (b) the IOP-29 is similarly valid when addressing feigning of different conditions such as depression, neuropsychological impairment, psychosis and/or PTSD (e.g., Giromini et al., 2020b; Ilgunaite et al., 2020; Winters et al., 2020); (c) the validity of the IOP-29 is maintained both when adopting a simulation/analogue (e.g., Gegner et al., 2021) and when relying on a known-groups comparison (Roma et al., 2020) research paradigm; (d) the IOP-29 yields incremental validity when used in combination with other SVTs (Giromini et al., 2019) or PVTs (Giromini et al., 2020a); (e) the IOP-29 preserves its effectiveness also when used outside the USA, in countries such as Australia (Gegner et al., 2021), the UK (Winters et al., 2020), Italy (Giromini et al., 2018), Portugal (Giromini et al., 2020a), or Lithuania (Ilgunaite et al., 2020).

## Overview of the Current Project

According to the test manual (Viglione & Giromini, 2020), the IOP-29 may be administered using either a paper-and-pencil or an online/computerized format. The latter is available (www.iop-test.com) for both in-person and remote administrations and prevents test-takers accessing the practitioner’s IOP-29 account and previous administrations. Nevertheless, so far, the great majority of published IOP-29 studies utilized a classic, in person, paper-and-pencil administration format. In fact, out of the 12 currently published IOP-29 studies referenced above, only two (i.e., Gegner et al., 2021; Winters et al., 2020) were conducted using a remote/online administration format. As such, additional research on the comparability and validity of the in-person and remote/online administrations of the IOP-29 would be beneficial.

The current project aimed at filling this gap in the literature, by conducting two separate studies. Study 1 compared the FDS values generated by three community samples that had been administered the IOP-29 remotely (*n* = 146), versus in-person, via computerized format (*n* = 140), versus in-person, via paper-and-pencil format (*n* = 304). Next, Study 2 conducted a mini-review of the literature aimed at determining the extent to which the two aforementioned IOP-29 studies that used a remote/online administration format (i.e., Gegner et al., 2021; Winters et al., 2020) yielded results comparable to those reported by other published IOP-29 studies that addressed similar research questions using an in-person format. More specifically, given that Gegner et al. (2021) investigated feigning of mTBI, Study 2 retrieved from the literature all other IOP-29 articles that addressed feigning of mTBI, and compared the validity results from these retrieved studies against those reported by Gegner et al. (2021). Along similar lines, because Winters et al. (2020) investigated feigning of schizophrenia, Study 2 retrieved from the literature all other IOP-29 articles that addressed feigning of schizophrenia and compared results from these studies against those reported by Winters et al. (2020).

It is worth noting that the research studies assembled for this article varied in their goals and methods, and none of them specifically aimed at examining the equivalence between face-to-face and remote administrations. Moreover, the majority were completed before the COVID-19 pandemic, when physical distancing was not required, and none used procedures recommended by experts for conducting adequate forensic teleassessment: for example, test-takers included in these studies were not asked to use a live video connection nor to share their screen during the administration of the tests. They simply received a link in which they were instructed to fill out the IOP-29 (and in some cases some other tests) online, without supervision. Therefore, the research setting used in the investigations examined by Study 1 and Study 2 was notably different from the typical, real-life, forensic teleassessment setting.

## Study 1: Equivalence of IOP-29 Scores from Different Administration Formats

Study 1 compared IOP-29 scores derived from 590 non-clinical individuals who had been administered the IOP-29 under standard instructions (i.e., with the request to respond honestly) in one of following the three formats: (a) online, from remote (*n* = 146); (b) in-person, via computerized format (*n* = 140); or (c) in-person, via paper-and-pencil administration (*n* = 304). The remote/online subsample came from an ongoing research study that was being conducted within the department of Psychology at the University of Turin when we initiated this project; the in-person/computerized subsample came from a study previously conducted by Pignolo et al. (under review); the in-person/paper-and-pencil subsample came from two previously published research articles: approximately half (*n* = 144) came from Giromini et al. (2020b); the other half (*n* = 160) came from Giromini et al. (2020d). In all cases, participants were Italian non-clinical adult volunteers who took the IOP-29 with the request to respond honestly to all its items.

### Method

#### Participants

Participants were 590 Italian adult individuals who volunteered for one of four different simulation studies aimed at testing the validity of the IOP-29. Ages ranged from 18 to 84, with an average age of 35.6 years (*SD* = 14.6); about half (53.5%) defined their gender as “female” and about half (46.5%) defined their gender as “male.”^1^ Additional information regarding their educational level, marital status, and other similar variables were available only partially, so they are not reported here. However, given that all studies from which this data set was generated used virtually identical recruitment procedures, it is highly likely that the characterization of our sample with regard to these variables closely resembles that reported in Giromini et al. (2020d, b) and Pignolo et al. (under review).

The three subsamples were not balanced with respect to age, *F*(2,578) = 37.936, *p* < 0.001. More specifically, Bonferroni-corrected post hoc test revealed that the in-person/computerized subsample (*M* = 42.2, *SD* = 15.9) was the oldest subsample, the remote/online subsample (*M* = 28.0, *SD* = 9.2) was the youngest one, and the in-person/paper-and-pencil subsample (*M* = 36.2, *SD* = 14.5) was located half-way between the other two subsamples. Also, the three subsamples were not balanced on gender either, chi^2^ = 20.806, *p* < 0.001. Examination of standardized residuals indicated that the percentage of females was significantly higher within the remote/online subsample (73.5%) than it was within the in-person/computerized (53.6%) and in-person/paper-and-pencil (47.0%) subsamples. Follow-up analyses, however, revealed that neither age, |*r*| ≤ 0.088, *p* ≥ 0.130, nor gender (dummy code), |*r*| ≤ 0.085, *p* ≥ 0.143, correlated with the IOP-29 FDS within any of the three subsamples under investigation. As such, the observed differences in age and gender distributions across subsamples should not have any meaningful influence on the main results presented below.

#### Procedures

All participants included in our data set were Italian native-speaking adult volunteers recruited via convenience and/or snowball sampling procedures. All signed an informed consent form prior to being enrolled as research participants. Additional details concerning the procedures used by each of the studies from which our data set was retrieved are reported below. All had previously been approved by the applicable Institutional Review Board(s).

#### Remote/Online Subsample

The remote/online subsample was retrieved from an online project (newly collected data, unpublished study) aimed at evaluating the extent to which a “naïve” participant would be able to identify the IOP-29 and a few other measures as “feigning tests.” Participants retrieved from that project and included in our remote/online subsample (*n* = 146) had originally been recruited as the “control group” of a simulation study. All were thus given a LimeSurvey link in which they were instructed to fill out a number of psychological tests online, with the request to respond as honestly as possible.

#### In-Person/Computerized Subsample

The in-person/computerized subsample was retrieved from another simulation study, which was originally designed to investigate the validity of the Personality Assessment Inventory (PAI; Morey, 1996, 2007) and IOP-29 in the detection of feigned psychopathology (Pignolo et al., under review). In that study, also conducted in Italy, the PAI and IOP-29 were administered to a community and forensic samples; about half were instructed to feign psychopathology, and about half were asked to respond honestly (control group). Within each sample, assignments of participants to the feigning or control groups were made on a random basis. The in-person/computerized subsample of the current study includes the 140 community-based individuals who were assigned to the control group and administered the IOP-29 in-person, via computerized administration.

#### In-Person/Paper-and-Pencil Subsample

The in-person/paper-and-pencil subsample was retrieved from two different research projects. As it was the case for the data collected from the two other subsamples, all participants included in this subsample also had been administered the IOP-29 under standard instructions, with the request to respond as honestly as possible.

A first subset (*n* = 160) was retrieved from Giromini et al. (2020d), in which the IOP-29 was administered along a few other measures to 360 nonclinical Italian volunteers—192 instructed to respond honestly (honest controls) and 168 instructed to feign mental illness (experimental simulators). Of the 192 participants included in the control group, 32 were elderly participants who were likely suffering from some cognitive impairment (many had previously experienced serious medical conditions such as ischemic strokes, tumors, or Parkinson’s disease), so that their data were not used for the current study.

The second subset of data we used to compile our in-person/paper-and-pencil subsample (*n* = 144) came from Giromini et al. (2020b). In this study, the IOP-29 was administered three times to 400 nonclinical Italian volunteers: in one condition, participants were asked to respond honestly (HON); in one condition, they were asked to feign a psychopathological condition (SIM); and in one condition, they were asked to respond at random (RND). Because the responses given to the cognitive items of the IOP-29 could change if the IOP-29 were taken multiple times by the same individual, we retrieved from Giromini et al.’s (2020b) data only the 144 cases in which the HON condition occurred first. These 144 IOP-29s, combined with the other 160 IOP-29s described in the previous paragraph, comprise our in-person/paper-and-pencil subsample (*n* = 304).

#### Data Analysis

The average IOP-29 FDS values produced by the three subsamples were compared with each other via a one-way ANOVA. Next, because null hypothesis significance testing (NHST) does not allow to provide support for the null hypothesis (*H*_0_), but only to prove that *H*_0_ cannot be accepted when that is the case (Altman & Bland, 1995), we also used Bayesian statistics. Specifically, we calculated Rouder et al.’s (2012) JZS Bayes Factor to estimate the relative posterior probability of the null (i.e., the three subsamples produce the same IOP-29 results; H0) and alternative (i.e., the three subsamples do not produce the same IOP-29 results; H1) hypotheses, given the data. This odds ratio was then interpreted based on Jeffreys’ (1961) criteria, according to which Bayes Factor values > 3, > 10, and > 30 are characterized, respectively, as “some evidence,” “strong evidence,” and “very strong evidence” for *H*_0_ over *H*_1_.

Next, we focused on classification accuracy and investigated whether the number of participants above versus below the standard IOP-29 cutoff score of FDS ≥ 0.50 (Viglione & Giromini, 2020) meaningfully differed across the three subsamples. To do so, first we computed a chi^2^ test; then we used procedures described by Gunel and Dickey (1974) to evaluate the independence assumption also via a Bayes factor, computed considering an independent multinomial sampling scheme (Jamil et al., 2017). This Bayes factor also was interpreted based on Jeffreys’ (1961) criteria summarized above.

### Results

The average IOP-29 scores produced by the three subsamples under investigation are reported in Table 1. They are strikingly similar to each other, and in fact, the ANOVA is not statistically significant, *F*(2,587) = 0.601, *p* = 0.549. More importantly, JZS Bayes Factor is equal to 27.031, indicating that the null hypothesis is almost 30 times more likely than the alternative, given the data. Based on Jeffreys’ (1961) criteria, thus, there is “strong evidence” (almost “very strong evidence”) that the administration format does not influence the IOP-29 FDS.

**Table 1:** IOP-29 results by administration format

		Remote/Online (*n* = 146)	In-Person/Computerized (*n* = 140)	In-Person/Paper-and-pencil (*n* = 304)	Entire sample (*N* = 590)
IOP-29 FDS values					
*M*		.172	.190	.185	.183
*SD*		.142	.148	.148	.147
IOP-29 classification accuracy					
IOP-29 FDS < .50		*n* = 140; 95.9%	*n* = 134; 95.7%	*n* = 289; 95.1%	*n* = 563; 95.4%
IOP-29 FDS ≥ .50		*n* = 6; 4.1%	*n* = 6; 4.3%	*n* = 15; 4.9%	*n* = 27; 4.6%

Underlined values denote specificity statistics

Table 1 also informs on the classification accuracy estimates calculated based on the standard IOP-29 cutoff of FDS ≥ 0.50 (Viglione & Giromini, 2020). Once again, the three subsamples yielded virtually identical results, with specificity ranging from 95.1 to 95.9%. The chi^2^ statistic is not significant, chi^2^ = 0.189, *p* = 0.910, and the associated Bayes Factor is 351.227. Based on Jeffreys’ (1961) criteria, thus, there is “very strong evidence” that the IOP-29 preserves the same specificity level when going from one administration format to another.

### Study 1: Discussion

The current study was undertaken to investigate the extent to which the IOP-29 scores obtained with a remote/online versus in-person/computerized versus in-person/ paper-and-pencil administration format would be equivalent to each other. Examination of 590 IOP-29 protocols from various ongoing and archival research projects revealed that the mean and standard deviation values generated by the three subsamples, as well as their associated specificity levels, were remarkably similar to each other. Bayesian statistics strongly confirmed this conclusion. Taken together, these findings suggest that administering the IOP-29 in-person or remotely and via paper-and-pencil or computerized format should yield the same or nearly the same results.

A few limitations, however, should be pointed out. First, our samples only included non-clinical, Italian individuals, so that the generalizability of our findings to other populations (e.g., different cultures, clinical cases) should be evaluated further. Second, we relied on archival data and convenience sampling and cannot demonstrate that all participants fully complied with the instructions to respond honestly to the IOP-29 items. To overcome this limitation, future studies are recommended to include validity checks or consider adding SVTs or PVTs. Third, the ecological validity is not a strength in that experimental findings might not generalize to high-stakes, real-life evaluations. Fourth, and perhaps more importantly, the fact that the scores generated by a honest community sample taking the IOP-29 via an in-person versus a remote/online formats are equivalent to each other does not necessarily guarantee that the validity of the IOP-29 will be preserved when going from one administration format to another. To that goal, it would be beneficial to also compare the effectiveness, classification accuracy, and validity results observed in other simulation/analogue studies in which the IOP-29 was administered in-person versus remotely.

## Study 2: Comparability of Published Validity Results

Study 2 was conducted to overcome some of the potential limitations of Study 1. More specifically, it consisted of a mini-review conducted to identify published studies that could inform on the comparability of the validity findings observed with the remote/online and in-person administration formats of the IOP-29.

### Articles’ Selection

As noted above, only two published studies have reported IOP-29 validity data derived from a remote/online administration format. Gegner et al. (2021) administered the IOP-29, its newly developed, optional, add-on, memory module (i.e., the IOP-M; Giromini et al., 2020d), and the Rey Fifteen-Item Test (FIT; Lezak, 1995; Rey, 1941) to an Australian community sample comprised of 275 volunteers. One third of the sample (*n* = 93) was asked to respond honestly and two thirds (*n* = 182) were instructed to feign mild traumatic brain injury (mTBI). Results strongly supported the effectiveness and validity of the IOP-29. Winters et al. (2020) also administered the IOP-29 from remote, but the focus of their study was on feigned schizophrenia, instead. More specifically, 151 British volunteers were administered the IOP-29 three times, under three different conditions, i.e., (a) responding honestly, (b) pretending to suffer from schizophrenia, and (c) responding at random. Also, in this case, results strongly supported the applicability and validity of the IOP-29.

Both the previous studies were simulation/analogue studies—one on mTBI (Gegner et al., 2021) and one on schizophrenia (Winters et al., 2020). Accordingly, only IOP-29 research publications that addressed these conditions via a simulation/analogue research paradigm were included in this mini-review. For each we present (1) the number of experimental simulators included in the selected study, (2) the number of individuals who took the IOP-29 under standard instructions (honest controls), (3) the characterization of the controls as presumably bona fide patients versus non-clinical/healthy individuals, (4) the administration language, (5) the sensitivity observed when using the standard a-priori IOP-29 cut-off of ≥ 0.50 (Viglione & Giromini, 2020), (6) the specificity observed when using the standard a-priori IOP-29 cut-off of ≥ 0.50 (Viglione & Giromini, 2020), (7) the Cohen’s *d* effect size obtained when comparing the credible versus non-credible groups, and (8) the Area Under the Curve (AUC) value associated with that same contrast.

### Results

Five research articles met the formal criterion for being included in this mini-review (i.e., being an IOP-29 article describing a simulation/analogue study on feigning of mTBI and/or schizophrenia). Three of them addressed both feigning of mTBI and feigning of schizophrenia, one informed on feigning of mTBI only and not on feigning of schizophrenia, and one informed on feigning of schizophrenia only and not on feigning of mTBI. All five research articles administered the IOP-29 in-person, via paper-and-pencil format.

#### IOP-29 Articles Addressing Feigned mTBI

Among retrieved articles, four reported information on the validity of the IOP-29 in the detection of feigned mTBI, when administered in-person. Each of them is briefly synthesized below.

#### Viglione et al. (2017)

In their original, developmental IOP-29 article, Viglione et al. (2017) utilized data from multiple sources and research projects, including a doctoral dissertation study by Pizitz (2001), in which the items of the IOP-29 were administered, in-person, to 38 adult volunteers instructed to feign mTBI and 38 individuals actually suffering from that disorder. About half of the data from these patients and feigners were used to scale the newly developed FDS; the other half was used for cross-validation purposes. For the summary findings described in this section focused on mTBI, we thus included the latter group only, which was comprised of 19 experimental simulators and 18 mTBI patient controls.

#### Giromini et al. (2020a)

Giromini et al. (2020a) administered the European Portuguese version of the IOP-29 along with the Test of Memory Malingering (TOMM; Tombaugh, 1996) to 100 adult Portuguese volunteers instructed to feign either depression (*n* = 50) or mTBI (*n* = 50). The current mini-review thus inspected the latter group only, comprised of 50 Portuguese feigners of mTBI.

#### Giromini et al. (2020b)

As mentioned above (see Study 1), Giromini et al. (2020b) administered the Italian version of the IOP-29 three times, to 400 nonclinical Italian volunteers, under three different conditions, i.e., (a) responding honestly (HON), (b) pretending to suffer from a psychopathological condition (SIM), and (c) responding at random (RND). In the SIM condition, different instructions were given to four groups of participants: 100 participants were asked to feign depression, 100 were asked to feign mTBI, 100 were asked to feign PTSD, and 100 were asked to feign schizophrenia. For the summary findings described in the section focused on mTBI of the current mini-review, we thus only examined the results coming from the HON and SIM conditions relative to the mTBI group.

#### Giromini et al. (2020d)

In this investigation, used also for Study 1, four independent research projects focused on feigning of four different conditions, i.e., neuropsychological problems (NP), depression, PTSD, or schizophrenia, were conducted. Within the NP subsample, 30 adult individuals aged < 70 and 32 elderly participants aged ≥ 70 were given the instruction to respond honestly, whereas 30 adult volunteers were asked to feign mTBI. The elderly subgroup was recruited as an example of individuals possibly characterized by cognitive impairment and indeed the majority of them scored relatively low on the Montreal Cognitive Assessment (MoCA; Nasreddine et al., 2005). All participants were from Italy and took the Italian version of the IOP-29. For the summary findings described in this mini-review section focused on mTBI, the IOP-29 results observed within these 92 participants were considered.

#### Summary of Results Concerning Feigned mTBI

Table 2 summarizes all relevant information concerning each of the studies on feigned mTBI included in this mini-review. When compared to all four studies conducted in-person using the paper-and-pencil version of the IOP-29, the sensitivity, specificity, Cohen’s *d* and AUC values reported by Gegner et al. (2021) were similar but consistently higher.

**Table 2:** Validity of the IOP-29 in detecting feigned mTBI: comparison between in-person and online administrations

	In-person administration				Online administration
	Viglione et al. (2017)^a^	Giromini et al. (2020a)^b^	Giromini et al. (2020b)^c^	Giromini et al. (2020d)^d^	Gegner et al (2021)^e^
Experimental simulators	19	50	100	30	182
Honest controls	18	-	100	62	93
Controls characterization	Patients	-	Healthy volunteers	Mixed sample	Healthy volunteers
Language	English	Portuguese	Italian	Italian	English
Se for IOP-29 FDS ≥ .50	0.84	0.88	0.88	0.87	0.96
Sp for IOP-29 FDS ≥ .50	0.94	-	0.94	0.94	1.00
Cohen’s *d*	2.37	-	3.27	2.83	5.31
AUC	0.93	-	0.96	0.95	1.00

^a^These data refer to Pizitz’s (2001) neuropsychologically injured subsample described in Study 2 of Viglione et al.’s (2017) article

^b^These data refer to the mTBI subsample described in Giromini et al.’s (2020a) article

^c^These data refer to the mTBI-related subsample of Giromini et al.’s (2020b) article: this study used a within-subject design, in which participants were asked to take the IOP-29 three times, one time answering honestly, one time faking mental illness, and one time responding with a random-like approach

^d^These data refer to the mTBI subsample described in Giromini et al.’s (2020d) article, which includes 30 honest adults, 32 honest elderly, and 30 simulators of mTBI

^e^These data refer to the 182 coached and uncoached mTBI simulators and 93 healthy controls described in Gegner et al.’s (2021)

#### IOP-29 Articles Addressing Feigned Schizophrenia

Four articles reported information on the validity of the IOP-29 in the detection of feigned schizophrenia, in its paper-and-pencil version. Each of them is briefly described below.

#### Viglione et al. (2017)

One of the several samples analyzed by Viglione et al. (2017) to cross-validate the newly developed FDS of the IOP-29 comprised 45 individuals with a diagnosis of schizophrenia or schizoaffective disorder and 45 experimental simulators—matched with patients on gender and level of education (Wood, 2008). These data were thus included in the summary findings of this mini-review section focused on schizophrenia.

#### Giromini et al. (2018)

This study administered the IOP-29 and SIMS to 452 Italian participants: 216 were individuals with mental illness who were asked to take both tests under standard instructions (control group), and 236 were experimental simulators. Within the control group, 89 individuals were suffering from a psychotic spectrum disorder and 127 were suffering from a non-psychotic, anxiety, depression, and/or trauma-related disorder. With regard to the simulator group, 125 were instructed to feign schizophrenia and 111 were instructed to feign depression, anxiety, and/or trauma-related symptoms. For the summary findings described in this section focused on schizophrenia, the IOP-29 results observed within the 125 feigners of schizophrenia and the 89 individuals suffering from a psychotic spectrum disorder were examined.

#### Giromini et al. (2020b)

As noted above, 100 of the participants included in Giromini et al. (2020b) were asked to take the IOP-29 three times, under three different conditions, one of which involved feigning schizophrenia. More specifically, in one condition, these 100 Italian participants were asked to respond honestly; in one to feign schizophrenia; and in one to respond at random. The IOP-29s coming from the honest and feigning conditions of this schizophrenia-related subgroup were thus included in the summary findings of this mini-review section focused on feigned schizophrenia.

#### Giromini et al. (2020d) 

As noted above, this article included four independent research projects focused on feigning of four different conditions, one of which addressed feigned schizophrenia. This schizophrenia-related subset included 45 experimental simulators and 40 non-clinical controls. Our mini-review thus included these data for the current section, focused on feigning of schizophrenia.

#### Summary of Results Concerning Feigned Schizophrenia

All relevant information concerning each of the IOP-29 studies focused on feigned schizophrenia and included in this mini-review is summarized in Table 3. The Cohen’s *d* effect size and AUC values reported by Winters et al. (2020) are slightly lower than those reported by Giromini et al. (2020d), but higher than those reported by the other three studies under consideration. Along similar lines, the sensitivity and specificity values found by Winters et al. (2020) are the second highest values across all studies.

**Table 3:** Validity of the IOP-29 in detecting feigned schizophrenia: comparison between in-person and online administrations

	In-person administration				Online administration
	Viglione et al. (2017)^a^	Giromini et al. (2018)^b^	Giromini et al. (2020b)^c^	Giromini et al. (2020d)^d^	Winters et al (2020)^e^
Experimental simulators	45	125	100	45	151
Honest controls	45	89	100	40	151
Controls characterization	Patients	Patients	Healthy volunteers	Healthy volunteers	Healthy volunteers
Language	English	Italian	Italian	Italian	English
Se for IOP-29 FDS ≥ .50	0.82	0.82	0.94	0.89	0.92
Sp for IOP-29 FDS ≥ .50	0.80	0.81	0.89	1.00	0.97
Cohen’s *d*	1.95	1.80	3.16	4.63	4.20
AUC	0.92	0.89	0.96	1.00	0.99

^a^These data refer to Wood’s (2008) psychotic subsample described in Study 3 of Viglione et al.’s (2017) article

^b^These data refer to the psychotic subsample described in Giromini et al.’s (2018) article

^c^These data refer to the schizophrenia-related subsample of Giromini et al.’s (2020b) article: this study used a within-subject design, in which participants were asked to take the IOP-29 three times, one time answering honestly, one time faking mental illness, and one time responding with a random-like approach

^d^These data refer to the schizophrenic subsample described in Giromini et al.’s (2020d) article

^e^These data refer to the schizophrenia-related subsample of Winters et al.’s (2020) article: this study used a within-subject design, in which participants were asked to take the IOP-29 three times, one time answering honestly, one time faking schizophrenia, and one time responding with a random-like approach

### Study 2: Discussion

Study 2 consisted of a mini-review of available IOP-29 literature possibly informing on the comparability of validity results obtained when administering the IOP-29 in-person versus from remote. Taken together, the results summarized in Tables 2 and 3 suggest that the validity of the IOP-29 should extend to the remote/online format with no loss in terms of classification accuracy and precision. In fact, both when inspecting feigned mTBI and when focusing on feigned schizophrenia, the studies that administered the IOP-29 online (from remote) yielded similar or perhaps even better validity results.

Study 2 also has several limitations that should be kept in mind when considering its practical implications. First, both Gegner et al. (2021) and Winters et al. (2020) used healthy, non-clinical volunteers, rather than bona fide patients, as controls, which is known to artificially boost specificity (Rogers & Bender, 2018; van Impelen et al., 2014). As such, to more fairly compare Gegner et al.’s (2021) and Winters et al.’s (2020) data against previously published studies, one should probably only look at investigations that included healthy volunteers as control groups, in Tables 2 and 3. Even so, however, the data obtained when administering the IOP-29 remotely versus in-person would still yield remarkably similar results. Second, a limitation of Study 2 is that all studies included in this mini-review used a simulation/analogue design, which limits ecological validity. Future research using a known-groups comparison paradigm are, therefore, needed to overcome this problem. Third, as we only focused on feigned mTBI and feigned schizophrenia, additional IOP-29 research on the comparability of validity findings obtained when investigating feigned PTSD or feigned depression would be beneficial.

## Overall Discussion

With this article, we sought to contribute to the ongoing debate on whether there is a significant departure from standard testing protocols when one performs teleassessment. More specifically, we focused on the IOP-29 and examined whether its average scores and classification accuracy estimates change when one uses a remote/online versus in-person administration format. Study 1 found that non-clinical controls taking the IOP-29 online from remote, in-person via computerized format, or in-person via paper-and-pencil generated virtually identical results. Study 2 further supported the hypothesis that the different administration formats are essentially equivalent to each other, by showing that published studies conducted using a remote/online versus in-person administration of the IOP-29 produced comparable validity findings for certain types of patients and feigners. Taken together, these findings suggest that administering the IOP-29 from remote should likely preserve the integrity of its psychometric properties.

A few concluding considerations and overall limitations deserve mention. In real-life, forensic tele-assessments, appropriate precautions should be taken to identify test-takers and their locations, to ensure that the testing session runs smoothly, without notable disruptions, etc. (Drogin, 2020). Unfortunately, the studies presented in this article did not take these precautions, as research participants in our online studies were simply invited to fill out the IOP-29 from remote and left on their own while delivering their responses. They were not monitored with the aid of a synchronous (live) teleconference applications, nor was an on-site proctor available to supervise the session. Thus, one might question whether our findings generalize to real-life evaluation contexts which use these precautions. On the other hand, inattentive item review or inconsistent effort would likely obscure discrimination of feigners from honest responders (Giromini et al., 2020c). Indeed, we had no control over the participants, so feigners might have searched the internet for the answers or asked family members and friends how to respond to the items, etc. Successful feigning in this context might be easier compared to feigning in a real-life setting, in which the examinee has to perform continuously in front of the examiner (either in-person or live) during the administration of tests. And yet, the IOP-29 demonstrated similar levels of discrimination in online and in-person conducted samples.

From a broader perspective, this article responds to the call to provide scientific evidence for the equivalence of remote versus face-to-face testing (Wright et al., 2020). Prior to our investigation, indeed, tests evaluating response styles and the credibility of presented symptoms had yet to prove their validity and reliability in the context of remote testing. Research on the MMPI instruments, for instance, have only focused, so far, on the equivalence between in-person via paper-and-pencil versus in-person via computer administration formats, and studies addressing a similar research question with other SVTs (e.g., Structured Interview of Reported Symptoms, SIRS-2; Rogers et al., 2010; Miller Forensic Assessment of Symptoms Test, M-FAST; Miller, 2001; Structured Inventory of Malingered Symptomatology, SIMS; Smith & Burger, 1997; Widows & Smith, 2005; Self-Report Symptom Inventory, SRSI; Merten et al., 2016) have yet to be published. With all due caution, our investigation thus provides some initial evidence that the validity of a self-report SVT like the IOP-29 may be expected to be preserved, when switching from a in person to an online/remote format.

## Footnotes

1 These statistics are based on valid data; however, age information was missing for nine cases, and gender information was missing for 52 cases.
